# Surging to strengthen emergency response

**DOI:** 10.2471/BLT.23.020123

**Published:** 2023-01-01

## Abstract

Five sub-Saharan African countries have launched initiatives to improve emergency response quality and timeliness. Tatum Anderson reports.

In November 2020, Dr Akawulu N'Djao received a call about a suspected cholera outbreak in southern Togo. Director of the Tchaoudjo health district in Togo, N’Djao heads a team responsible for confirming disease outbreaks and implementing response measures.

“We investigated in collaboration with colleagues from Sotouboua and Ogou districts,” he recalls. “When we arrived in the village, we learned that a woman had died displaying cholera symptoms two weeks earlier. By the time we got there, cases were exploding. If the first case had been reported that would not have happened.”

It is a scenario played out across sub-Saharan Africa – and in many other parts of the world – on a regular basis. “It can take weeks to report cases and pull together human and material resources and get them to where they are needed,” says Dr Fiona Braka, the World Health Organization’s (WHO) emergency operations team leader based in Brazzaville, Congo. “This is a problem because the first 72 hours of any outbreak are critical. Beyond that time the challenges and the fatalities may start to increase.”

In the worst cases, response times are measured in months. This was true of the response to an outbreak of cholera which began in the Dosso and Tahoua regions in Niger in March 2021, for example.

“The outbreak was not reported until August at which time response efforts were initiated,” recounts Dr Bienvenu Baruani, an emergency preparedness officer at WHO’s country office in Niamey, Niger. “By the time the outbreak was brought under control, cholera had reached all seven regions of the country, with around 3000 cases in Zinder Region alone. Up to 150 people died.”

Emergency response quality and timeliness is coming under increased scrutiny as countries struggle with an ever-increasing burden of emergencies, much of it related to climate change. “Countries across the African continent face over 100 public health emergencies every year, around 80% of them related to infectious diseases,” Braka explains. “A further 20% are driven by floods, famine, and conflict.”

Ongoing emergencies include those unfolding in the Horn of Africa where many regions are battling the worst drought in at least 40 years.

Countries that have seldom seen adverse weather events in the past are also being affected. “Botswana has never been considered a particularly disaster-prone country,” says Maeletso Pego of the National Disaster Management office in Gaborone, Botswana. “But climate change has brought with it a multitude of problems that threaten the country’s food production. Our rainy season is now erratic, and when the rains do come, they cause widespread flooding.”

“Climate change has brought with it a multitude of problems.”

Maeletso Pego

Strengthening emergency response was a focus of discussions at the Seventy-second session of the WHO Regional Committee for Africa which took place in Lomé, Togo in August 2022. The session ended with health ministers adopting a new eight-year strategy designed to promote and support preparedness and surveillance systems and boost emergency response.

Ministers also agreed to 12 targets for 2030, including at least 90% of African Member States being able to mobilize an effective response within 24 hours of an emergency. This is to be achieved through implementation of the Strengthening and Utilizing Response Groups for Emergencies (SURGE) initiative.

The value of SURGE is already being demonstrated, five sub-Saharan countries (Botswana, Mauritania, Niger, Nigeria and Togo) having launched SURGE initiatives in the first half of 2022.

As the acronym suggests, SURGE is focused on the rapid deployment of people and equipment when emergencies arise. Core to its implementation are rapid-response teams comprised of roughly 50 specialists in disciplines ranging from epidemiology and infectious disease control to communications and logistics. The teams are coordinated by in-country Emergency Operations Centres (EOCs) staffed with designated public health emergency management personnel.

As Braka points out, EOCs have been a part of the emergency response landscape in Africa for some time and 37 Member States already have one. However, they have not always been properly resourced or effectively implemented within emergency response systems, reflecting broader disparities in emergency response capacity. The SURGE initiative is an opportunity to address that problem, among others.

WHO is mobilizing $2 million United States dollars in catalytic support for each target country over the first two years, to train the SURGE teams, facilitate their deployment during emergencies and buy the emergency vehicles and other equipment needed to carry out their tasks. WHO is also facilitating systematic onboarding of SURGE staff and ongoing training in all aspects of response in disciplines ranging from logistics to incident management.

To ensure that capacity reflects local needs and challenges, training is being delivered by experts from African countries – a context-specific approach that is widely appreciated. “We get training from people from countries facing the same challenges we do or might face in the future,” says Pego, referencing a module on Ebola which he found particularly useful. “We have had no cases of Ebola to date but cannot rule out the possibility of experiencing such outbreaks.”

Drills and simulations are also being used to anchor training in reality. Dr Kokou Tossa, National SURGE and Public Health EOC Coordinator at Togo’s Ministry of Health, reports on table-top simulation exercises and drills focused on transporting infected people from border crossing points to hospital or case management centres. “We have many borders in our country, and the threat of cross-border outbreaks is very real,” he explains.

Togo already had a 250-strong rapid-response team in place – as well as an EOC – and its SURGE work has focused on strengthening the coordination of its response efforts. For Tossa, bringing together committed responders is vital. “It is not enough to just designate members of the team,” he explains. “Members need to be enthusiastic and committed. You also need to ensure that SURGE members’ contracts (most are employed in government departments) make it possible for them leave their posts when it is time to respond to an emergency.”

Capacity to work and coordinate with outside partners is also crucial. To date emergency response has generally been headed by international and regional agencies. 

“Some partners have an agenda, so to get them to work together and to work with them can be difficult,” says Dr Abdoulaye Yam, a former SURGE coordinator for Togo who is now working with the WHO Regional Office for Africa emergency preparedness unit.

“We want to see this embedded in the workings of the government.”

Fiona Braka

One of the biggest challenges to date has been mobilizing and sustaining SURGE funding. As noted, WHO is providing catalytic support, but Braka underlines the fact that it will only last for two years and is meant to kick-start initiatives that will require more funding in the future.

“That’s why, when we go to countries, we meet the donors and partners in country, but also advocate for the government to allocate domestic resources to address the plan,” she says. “We want to see this embedded in the workings of the government, and not seen as a separate project.”

WHO Regional Office for Africa Regional Emergencies Director, Dr Salam Gueye, also emphasizes the need for national level engagement and is encouraged by the momentum that is building around the SURGE initiatives at country level, seeing them as an opportunity for system-level reform. “SURGE has the potential to unlock existing systemic challenges through African-driven solutions,” he says.

Braka, too, is optimistic and believes that progress is already being made. “It used to take an average of 17 days to detect an outbreak in sub-Saharan Africa and that is now down to just two days in most settings,” she says.

That was how long it took to respond to the most recent cholera outbreak in Niger, a marked improvement on the months it took in 2021. “After SURGE training and implementation, things really improved,” says WHO’s Bienvenu Baruani. “When cholera first appeared in Maradi region in September 2022, it was confirmed overnight using laboratory cultures. The team was in the field 48 hours after the first confirmed case.”

The SURGE team then organized case management, reinforcement of infection prevention and control and communication of infection risk to the communities concerned. A *cordon sanitaire* was set up around the cases, the drinking water was treated with tablets, people in the villages were reminded to wash hands and not to stay at home but to go to a treatment centre if they had symptoms. Oral rehydration was provided for those on their way for treatment to prevent the worsening of cases. The team put in place a cholera treatment centre and assessed the number of doctors and nurses available and relayed this back to the EOC. When it became clear the existing staff would not be enough to cope with the outbreak, the team reached out to Médecins Sans Frontières, an existing member of the SURGE coordination team, for support.

“By the time the last case was reported on October 16, there had been around 70 cases in just a few districts of two regions – Zinder and Maradi,” says Baruani. “There were only two deaths.”

**Figure:**
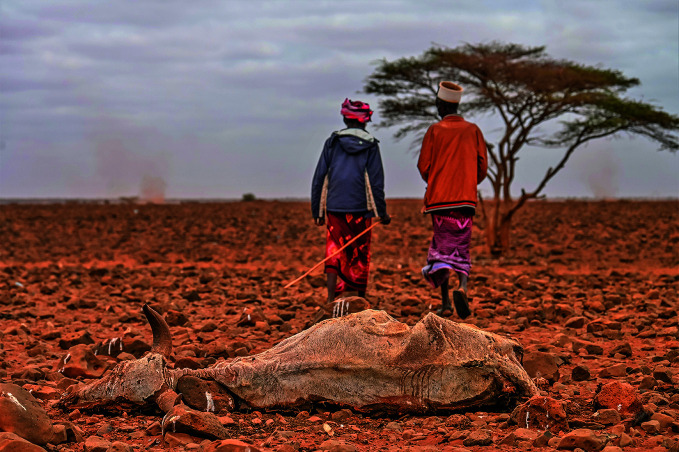
Men walk past a carcass in drought-stricken Marsabit County, Kenya.

WHO/Billy Miaron

**Figure:**
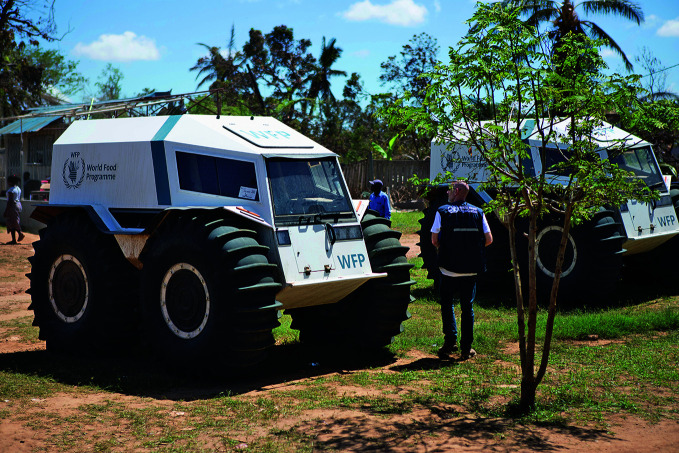
Trucks brought in to help repair Buzi District Hospital in Mozambique after Cyclone Idai.

WHO/Mark Nieuwenhof

